# Functional outcomes of full-endoscopic spine surgery for high-grade migrated lumbar disc herniation: a prospective registry-based cohort study with more than 5 years of follow-up

**DOI:** 10.1186/s12891-020-03891-1

**Published:** 2021-01-09

**Authors:** Christopher Wu, Ching-Yu Lee, Sheng Chi Chen, Shao-Keh Hsu, Meng-Huang Wu

**Affiliations:** 1 College of Medicine, Taipei Medical University, Taipei, Taiwan; 2 Department of Orthopaedics, School of Medicine, College of Medicine, Taipei Medical University, Taipei, Taiwan; 3 Department of Orthopedics, Taipei Medical University Hospital, R.O.C, No. 252,Wuxing St., Xinyi Dist., Taipei, 11031 Taiwan; 4 Department of Orthopedics, Tungs’ Taichung MetroHarbor Hospital, No.699, Sec. 8, Taiwan Blvd., Taichung City, 435 Taiwan

**Keywords:** Discectomy, Full-endoscopic lumbar discectomy, Migrated disc herniation

## Abstract

**Background:**

Full-endoscopic lumbar discectomy (FELD) is an alternative to posterior open surgery to treat a high-grade migrated herniated disc. However, because of the complexity of the surgery, success is dependent on the surgeon’s skill. Therefore, patients are frequently treated using open discectomy. Anatomical constraints and technical difficulties can lead to the incomplete removal of high-grade migrated discs.

**Methods:**

We retrospectively reviewed patients who had undergone FELD performed by a single surgeon between January 2010 and January 2014 from a prospective spine registry in an institute. Perioperative records and data of the Oswestry Disability Index, visual analog scale scores (preoperatively and 2 weeks, 6 weeks, 3 months, 6 months, 1 year, 2 years, and 5 years after the operation), and MacNab criteria were collected.

**Results:**

Of 58 patients with a follow-up duration of > 5 years, (41 and 17 patients had undergone transforaminal endoscopic lumbar discectomy [TELD] and interlaminar endoscopic lumbar discectomy [IELD], respectively), the satisfaction rate was 87.8% (five unsatisfactory cases) for TELD and 100% for IELD. The overall percentage of patients with good to excellent results according to modified MacNab criteria was 91.3% (53/58 patients). Two patients had residual discs. Two patients needed an open discectomy due to recurrent disc herniation. One IELD patient received spinal fusion surgery due to segmental instability after 5 years.

**Conclusion:**

FELD has a high success rate for the management of high-grade migrated herniated discs. In patients with high-grade disc migration from L1 to L5, TELD is effective and safe. However, for L4–L5 and L5–S1 high-grade upward and downward disc migration, IELD is the favorable option and provides high patient satisfaction.

## Background

In 1975, Hijikata described the first percutaneous discectomy; since then, full-endoscopic lumbar discectomy (FELD) has been frequently used for managing lumbar disc herniation [1, 2]. This alternative to conventional open discectomy has many benefits, such as decreased tissue trauma; lower postoperative instability; no interference with the epidural venous system, which, if damaged, may result in fibrosis and chronic neural edema; and faster recovery [3, 4].

Although FELD has many advantages, the indication for its use is mostly observed in patients with nonmigrated or low-grade migrated disc herniation. The incidence of migrated discs is approximately 35–72%, and most patients have a downward low-grade migrated disc (30.9%) [5, 6]. However, high-grade migrated discs are commonly observed (an incidence of 34% for migrated discs) [6, 7]. Because of the high failure rate of FELD in high-grade migrated disc herniation, open surgery is usually suggested; moreover, FELD is usually difficult because of anatomical barriers encountered when removing high-grade migrated discs, which can result in the incomplete removal of the disc material [4, 8].

Recently, the development of instruments and techniques has enabled the use of FELD to correct high-grade migrated lumbar discs. Many spine surgeons have developed novel techniques for managing high-grade migrated lumbar disc herniation by using FELD, including expanding the entry point of the transforaminal endoscopic lumbar discectomy (TELD) approach by using the foraminoplastic technique [4, 9–11], the transfacet process and pedicle-complex approach [12], two-level TELD [8], contralateral TELD [13], the suprapedicular approach [14], and the transpedicular approach [15, 16]. Alternatively, surgeons may opt to use a technique involving the posterior route, including the translaminar approach [17, 18], the interlaminar endoscopic lumbar discectomy (IELD) approach [19, 20], or adjacent IELD [21], which are similar to open surgery. Although improvements in equipment and techniques have resulted in better outcomes in FELD than in conventional open surgery, the management of high-grade migrated discs remains a challenge. In this study, we examined the long-term outcomes of patients with high-grade migrated disc herniation treated using FELD, and we reviewed the literature for the analysis of relevant surgical techniques.

## Methods

### Patients and evaluation

**Fig. 1:**
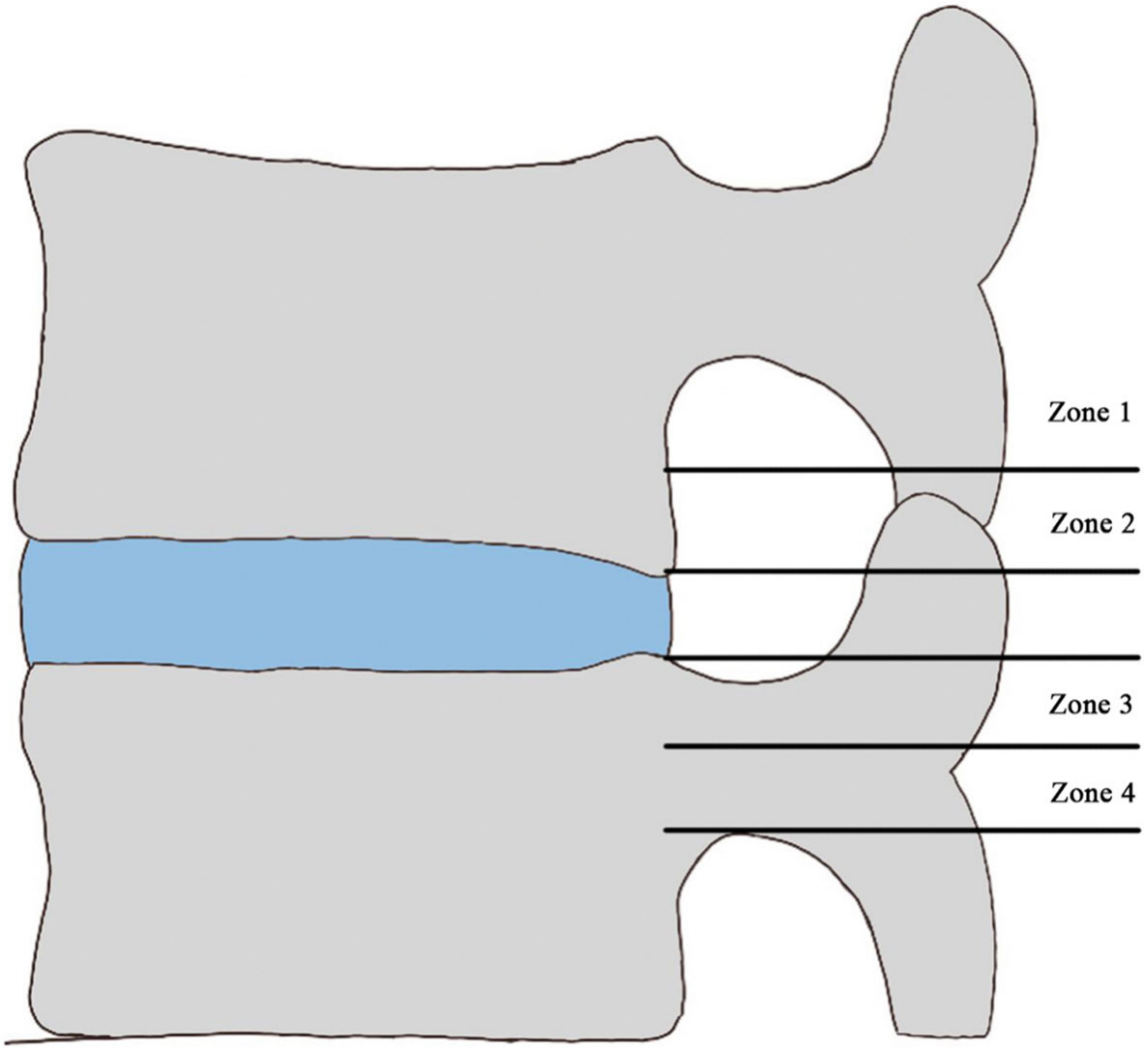
Radiological classification of migrated disc herniation

We retrospectively reviewed patients who had received FELD by a single surgeon between January 2010 and January 2014 from a prospective spine registry in an institute. Data concerning patients’ age, sex, and treatment time as well as follow-up data were collected. The computed tomography (CT) and magnetic resonance imaging (MRI) scans of patients were used to determine the level and extent of pathology. An intraoperative fluoroscopy examination was performed to ensure the correct positioning of the endoscope. The successful removal of migrated discs was determined using intraoperative findings (dural pulsation, loose neural element, retrieved disc fragments, and intraoperative symptoms) and postoperative symptoms. Herniated discs were classified using the radiological classification of migrated disc herniations provided by Lee et al. [22] (Table 1, Fig. 1). Migration into zone 1 and zone 4 was considered high-grade migration.

**Table 1:** Radiological Classification of Migrated Disc Herniation [22]

Zone	Direction	Range of Distance
Zone 1	Far upward	From the inferior margin of the upper pedicle to 3 mm below the inferior margin of the upper pedicle
Zone 2	Near upward	From 3 mm below the inferior margin of the upper pedicle to the inferior margin of the upper vertebral body
Zone 3	Near downward	From the superior margin of the lower vertebral body to the center of the lower pedicle
Zone 4	Far downward	From the center to the inferior margin of the lower pedicle

### Exclusion criteria

Patients were excluded if they had spinal stenosis confirmed through CT or MRI; exhibited segmental instability; exhibited other pathological conditions, such as acute inflammation, infection, fractures, or tumors; or were lost to follow-up within 5 years. This study was approved by our institutional review board (TMU-JIRB No.: N201903139).

### Surgical technique

#### Surgical position

During surgery, each patient was placed in a prone position to allow hip flexion to increase the available working space. This position reduces lordosis, allowing easy access to the spine.

#### TELD

**Fig. 2:**
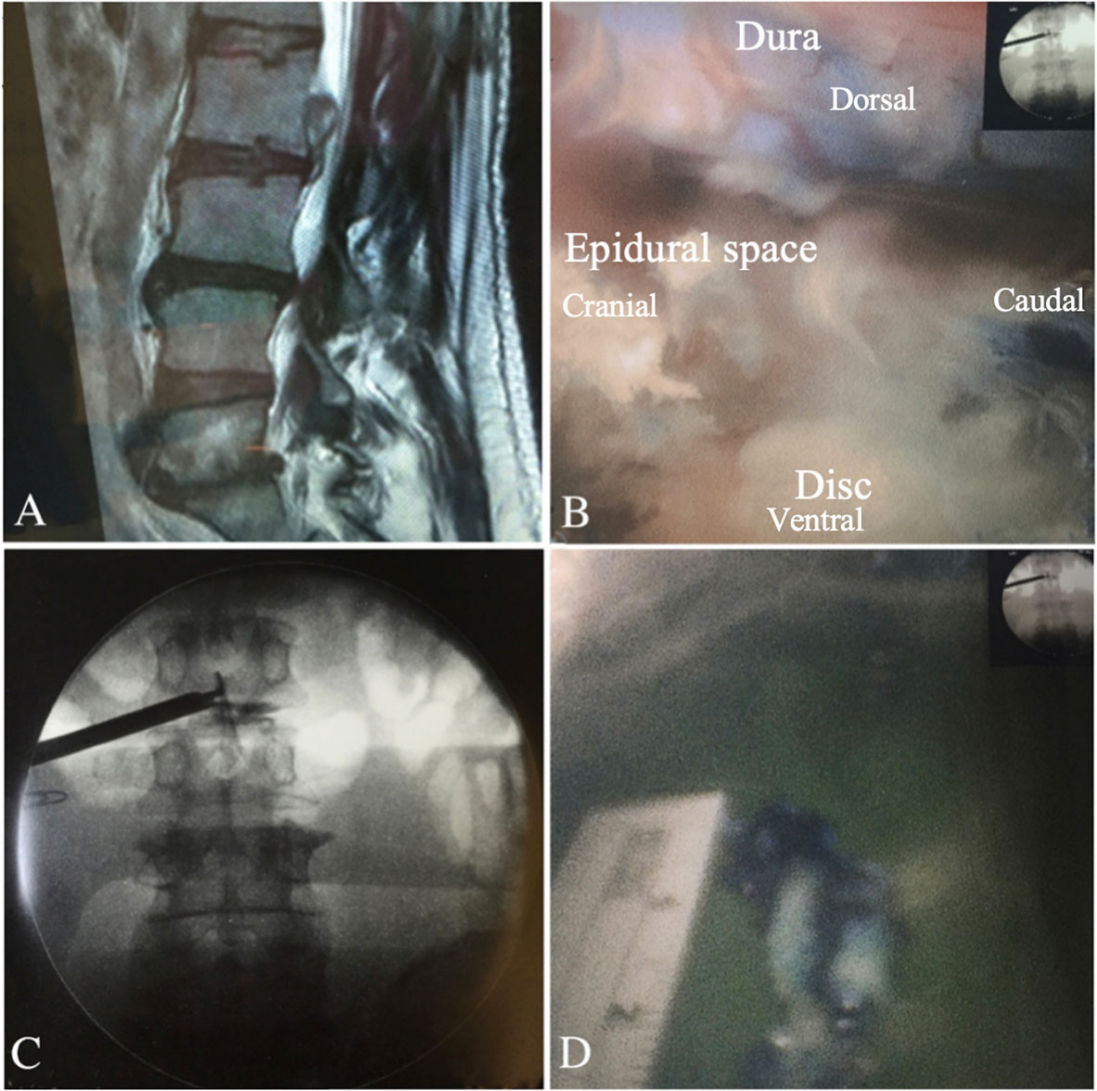
L2–L3 transforaminal full-endoscopic lumbar discectomy (TELD) for L2–L3 high-grade upward migration disc at zone 1. **a** Preoperative magnetic resonance imaging (MRI) lateral view showing an L2–L3 high-grade upward-migrated disc. **b** Intraoperative endoscopic view showing the L3 traversing root and epidural space. **c** The use of a flexible probe tip to pull out the migrated disc near zone 1 of the L2 vertebra. **d** Removed blue-stained migrated disc fragment

**Fig. 3:**
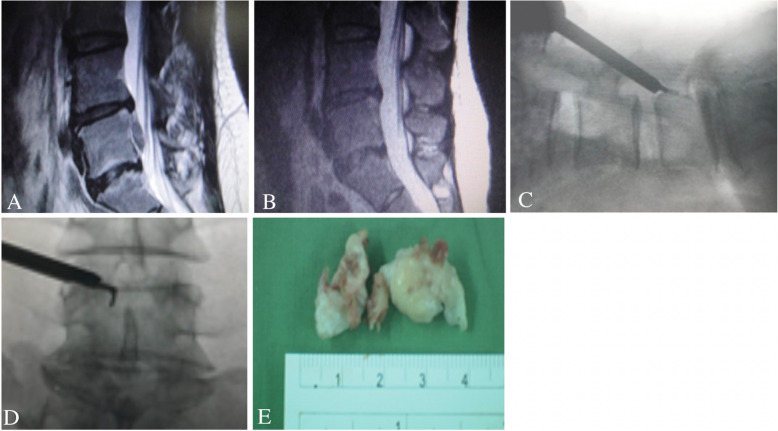
TELD for L4–L5 high-grade downward migrated herniated disc at zone 4. **a** Preoperative MRI lateral view showing an L4–L5 downward migrated disc. **b** Postoperative MRI lateral view showing complete decompression after the removal of the migrated disc. **c** and **d** Intraoperative fluoroscopy view showing flexible dissecting forceps probing down-migrated disc fragments at zone 4. **e** Removed disc fragments

**Fig. 4:**
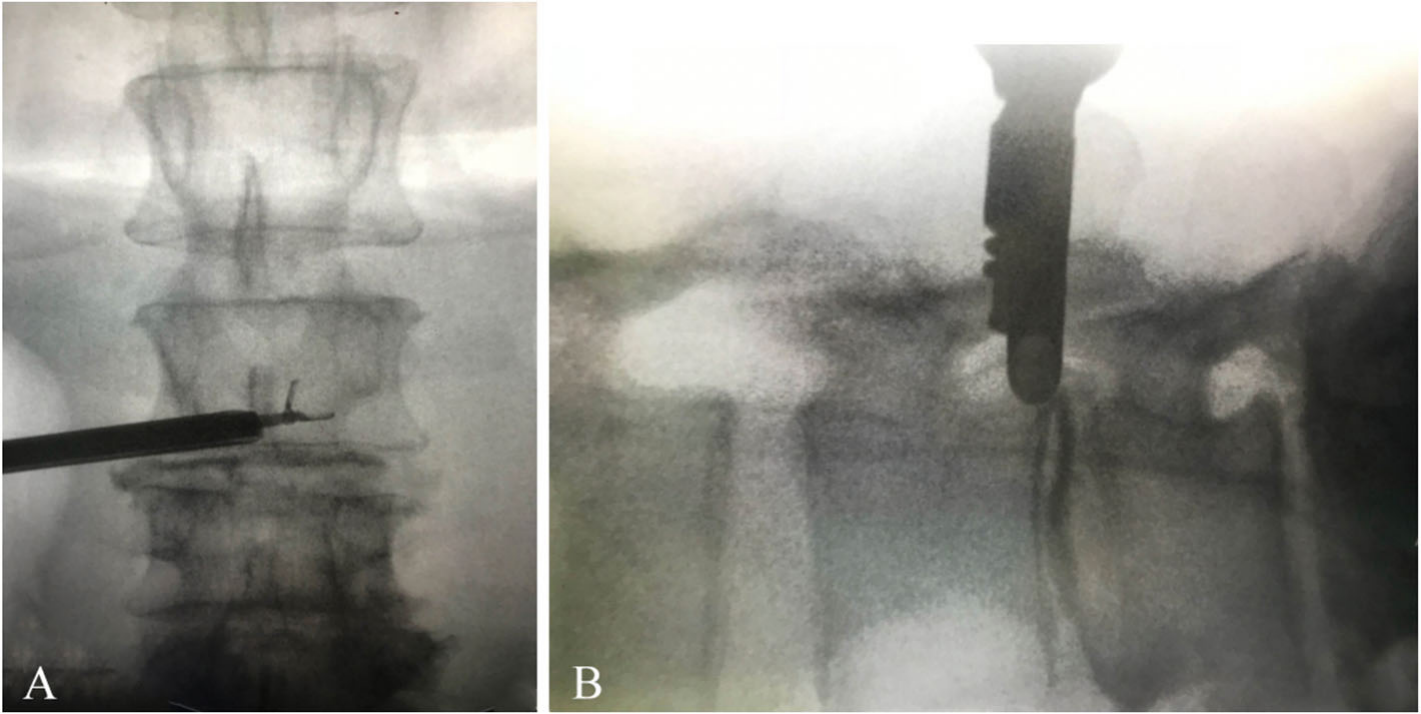
Contralateral TELD for upperward migration of L3–L4 HIVD at zone 1. **a** Anterior to posterior view of intraoperative fluoroscopy showing the endoscopic micro rongeur forceps grasping the disc fragment at contralateral zone 1. **b** Lateral view of the intraoperative fluoroscopy working channel position located at the epidural space

For high migration at the L1–L2 to L4–L5 level, the transforaminal epiduroscopic approach was selected (Figs. 2, 3, and 4). Patients were locally anesthetized using 1% lidocaine. Before starting the surgery, the patient was placed in a prone position. To establish the entry site, preoperative imaging studies along with intraoperative fluoroscopy were conducted. The skin entry depended on the patient and was generally 8–15 cm lateral from the midline. The approach angle for the disc depended on the direction and zone of the disc location. If the disc exhibited upward migration, then a caudal to cranial approach angle was selected, whereas if the disc exhibited caudal migration, then a cranial to caudal approach was adopted. Methylene blue dye was injected into the disc space to visualize the leakage pattern in the annular fissure. Dilatation was subsequently performed, and endoscopic exploration was initiated. Intradiscal subannular debulking was performed until the border of the annular fissure was uncovered. The outer layer of the annulus and the posterior longitudinal ligament were cut using a pair of annulus scissors. Once the outer annulus and posterior longitudinal ligament were cut, the epidural layer was released after the confirmation of the epidural space and the fragment of the migrated disc. This ventral decompression was expected to create additional working space to approach the disc that had migrated in the cranial or caudal direction. The herniated disc was observed after completing the epidural and intradiscal release. A pair of flexible forceps was used to remove the tip of the migrated disc under endoscopic and fluoroscopic guidance. The disc could be removed in one piece or in multiple pieces. Next, complete herniotomy was conducted by removing the entire herniation along with the intradiscal fragment, periannular fragment, and fragment that had migrated from the site. For two L3–L4 upward-migrated discs, contralateral TELD was used.

#### IELD

**Fig. 5:**
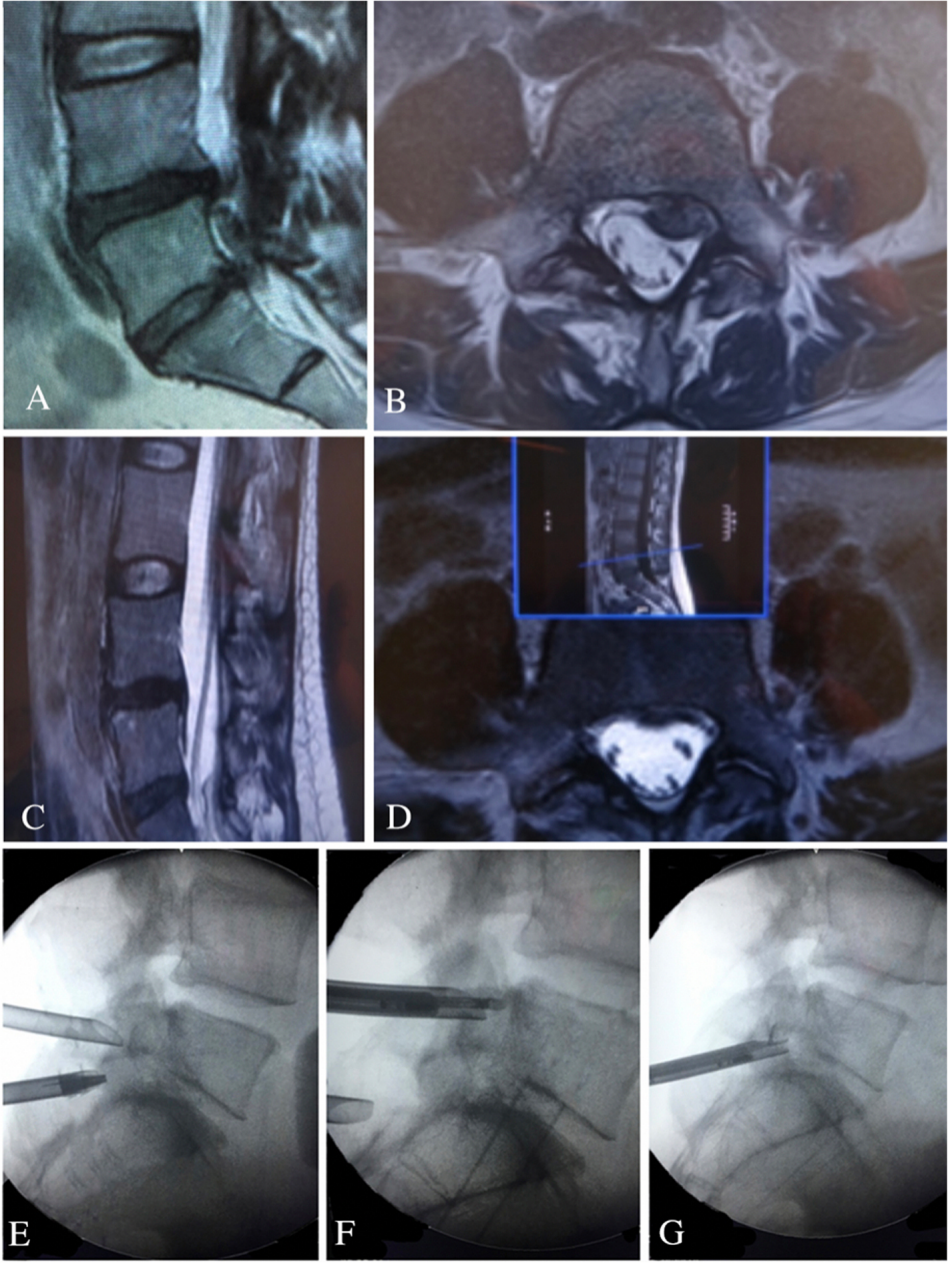
L4–L5 and L5–S1 interlaminar full-endoscopic lumbar discectomy (IELD) for L4–L5 high-grade downward migrated disc at zone 4 due to difficulty reaching the migrated fragment from L4–L5 IELD. **a** Preoperative MRI lateral view showing L4–L5 high-grade downward migration. **b** Preoperative MRI axial view showing L4–L5 herniated disc. **c** Postoperative MRI lateral view showing removal of the migrated disc. **d** Postoperative MRI axial view showing the removal of the migrated disc. **e** Intraoperative fluoroscopic view of the two working channels of the double IELD approach. **f** Intraoperative fluoroscopic view showing IELD from the L4–L5 interlaminar window for L4–S1 zone 4. **g** Intraoperative fluoroscopic view showing IELD for L5–S1 zone 1

**Fig. 6:**
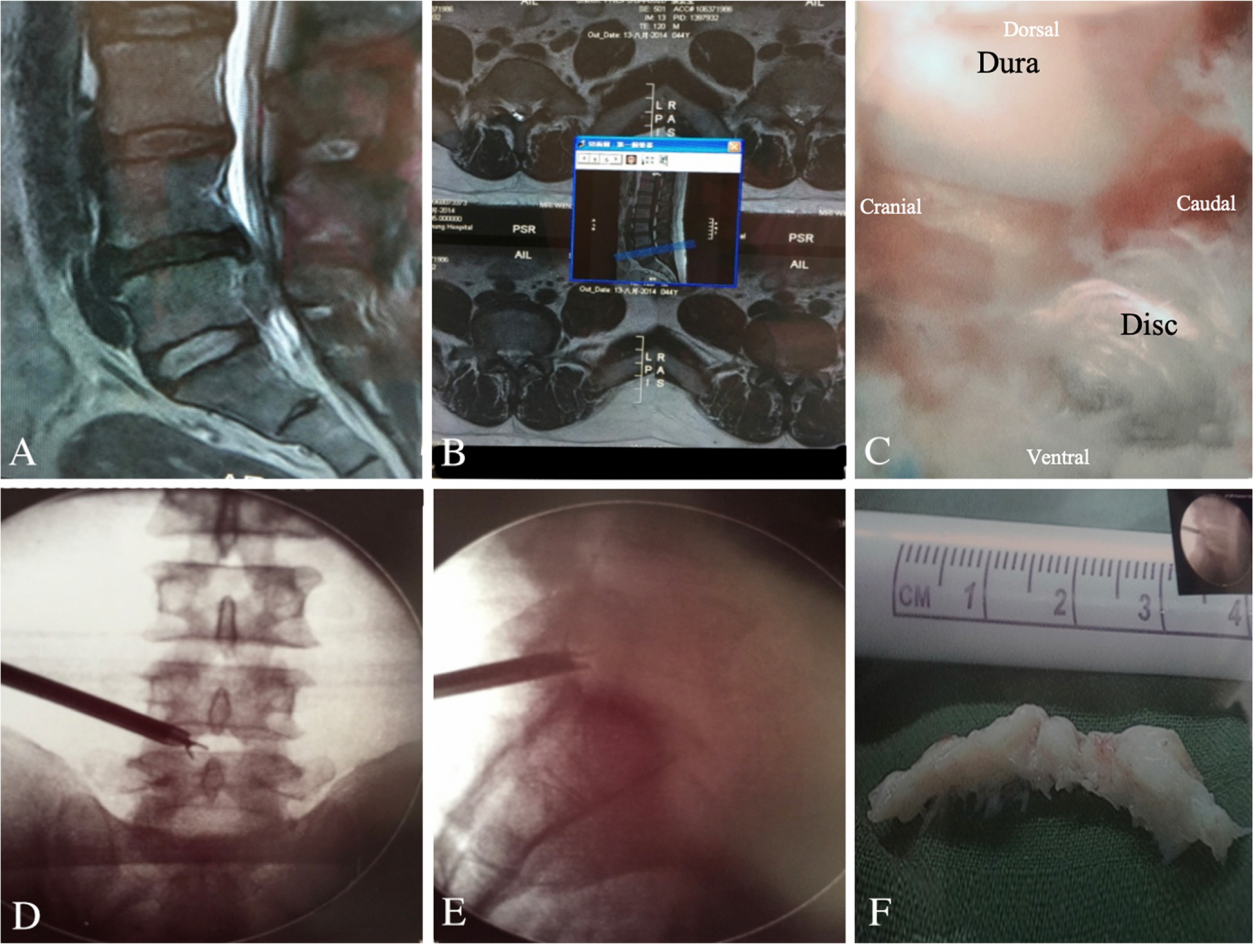
L4–L5 TELD changed to L5–S1 IELD for L4–L5 high-grade downward migration. **a** Preoperative MRI lateral view showing high-grade downward migrated disc herniation at the L4–L5 level. **b** Preoperative MRI axial views showing disc herniation at the L4–L5 level. **c** Intraoperative fluoroscopic view of the herniated disc material. **d** Fluoroscopy anteroposterior view showing forceps near the disc fragment during L4–L5 TELD. **e** Fluoroscopy lateral view showing the forceps grasping the disc fragment during L5–S1 IELD. **f** Removed disc fragments

**Fig. 7:**
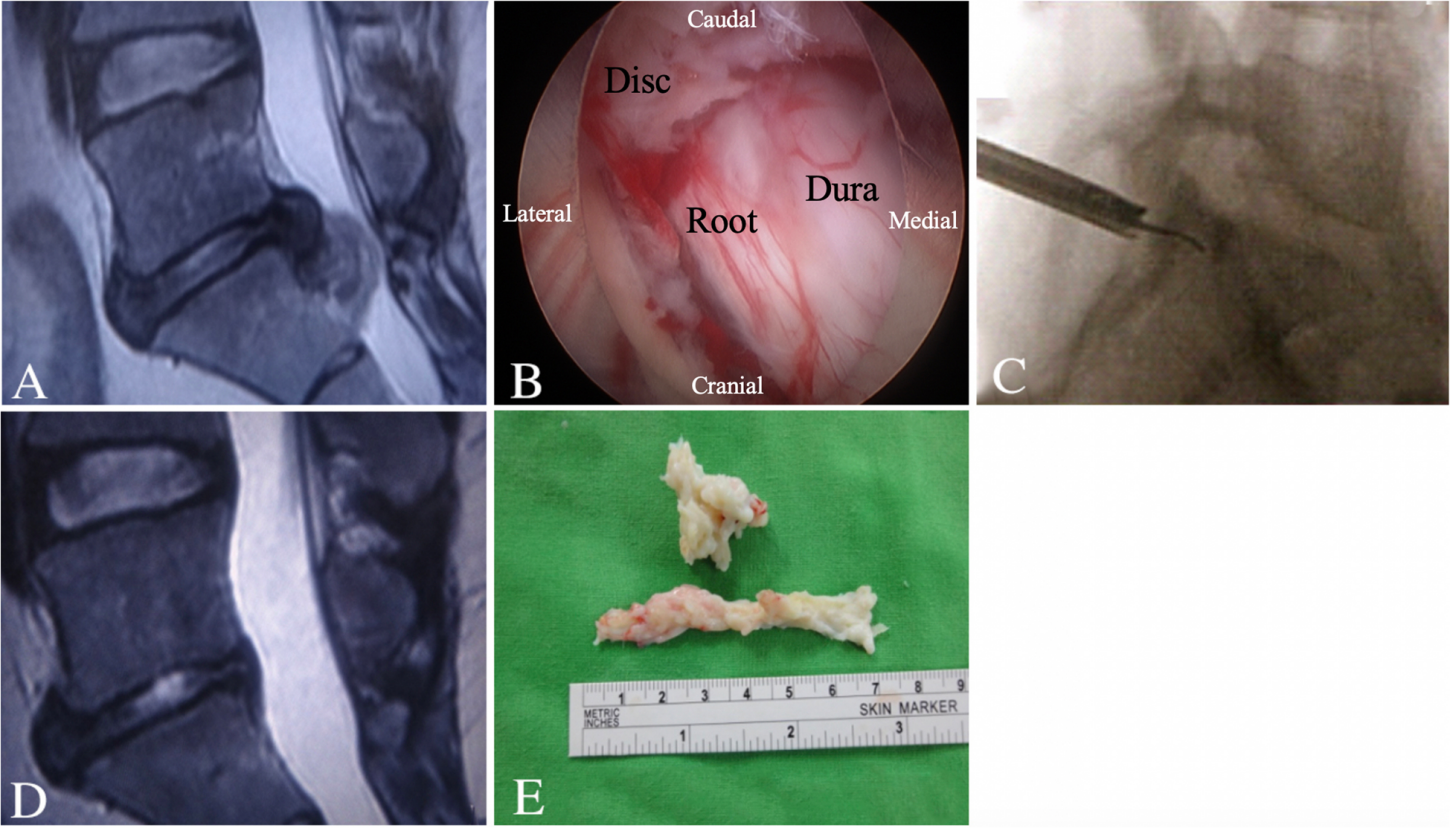
L5–S1 IELD for L5–S1 high-grade downward migration. **a** Preoperative MRI lateral view showing L5–S1 high-grade downward migration. **b** Intraoperative endoscopic view of the migrated disc. **c** Intraoperative fluoroscopic image of the probe at a high-grade migrated disc. **d** Postoperative MRI lateral view of the removed migrated disc. **e** Removed disc

IELD was performed in patients with L4–L5 high-grade downward disc migration or L5–S1 disc migration (Figs. 5, 6, and 7). The surgery was performed under general anesthesia. Patients were placed in the prone position, with their hips flexed to increase the interlaminar space. Soft tissue expanders were used to separate muscles to facilitate the insertion of the cannula and endoscope. The inferior edge of the cranial lamina on the side of the lesion and the ligamentum flavum (LF) were exposed using the endoscopic camera. To gain access to the spinal canal, a small incision was created on the LF by using a laminectomy rongeur. For L4–L5 discectomy, a variable drill was used to resect the cranial lamina to enlarge the interlaminar space. For L5–S1 discectomy, the spinal canal was exposed after dissecting the LF. A drill was used in some cases with narrow interlaminar space, such as L5–S1, to create an area easier to work in. Finally, the exposed herniated nucleus pulposus was removed to decompress the nerve root. In one patient with an L5–S1 upward migration disc, an L4–L5 and L5–S1 biportal-IELD was chosen.

#### Statistical analysis

GraphPad Prism 5 (La Jolla, CA, USA) was used to assess data. Data are expressed as the mean and range. Multiple *t* tests were performed to assess functional outcomes. To compare baseline demographic data between IELD and TELD, the chi-square value was calculated. The *t* test was also used to analyze differences between continuous variables such as the length of stay and operation time. Significance was set at *P* < 0.05 for all the tests.

## Results

### Patient demographic and perioperative data

A total of 68 patients with single-level high-grade migrated discs were enrolled in this study, and 58 patients were followed up for > 5 years (Table 2). In total, 41 and 17 patients underwent TELD and IELD, respectively. One patient received biportal-IELD for L4–L5 high-grade downward migration (Fig. 5). The operated levels included L1–L2 (1 patient, 1.47%), L2–L3 (4 patients, 5.88%), L3–L4 (6 patients 8.82%), L4–L5 (36 patients, 67.65%), and L5–S1 (11 patients16.18%; Table 2). The average follow-up duration was 6.1 years (range: 5.1–9.2 years). No difference in patient demographics or length of stay was observed between the IELD and TELD groups. However, the TELD group had more patients with zone 1 migration in proximal-level disc herniation (*P* = 0.032) and shorter operation time (*P* = 0.045) than did the IELD group.

**Table 2:** Demographic and Clinical Characteristics of Patients

	Overall	TELD	IELD	*P*
Overall	58	41	17	
Age (years)	56.3 (18–78)	56.7 (18–72)	55.2 (18–78)	0.462
Sex	38F 20M	27F 14M	11F 6M	0.332
ASA				0.175
1	32	24	8	
2	15	10	5	
3	11	6	5	
Zone 1				0.032
L1–L2	1	1	0	
L2–L3	4	4	0	
L3–L4	4	4	0	
L4–L5	10	8	2	
L5–S1	7	0	7	
Zone 4				0.511
L1–L2	0	0	0	
L2–L3	0	0	0	
L3–L4	2	2	0	
L4–L5	26	21	5	
L5–S1	4	1	3	
Operation duration (minutes)	55.7 (28–128)	50.6 (28–85)	65.5 (45–128)	0.045
Length of stay (days)	5.2 (3–7)	4.6 (3–6)	5.8 (3–7)	0.082
Follow-up (years)	6.1 (5.1–9.2)	6.3 (5.1–7.8)	5.7 (5.5–9.2)	0.617

*ASA* American Society of Anesthesiologists classification, *IELD* Interlaminar endoscopic lumbar discectomy, *TELD* Transforaminal endoscopic lumbar discectomy

### Functional outcomes

**Fig. 8:**
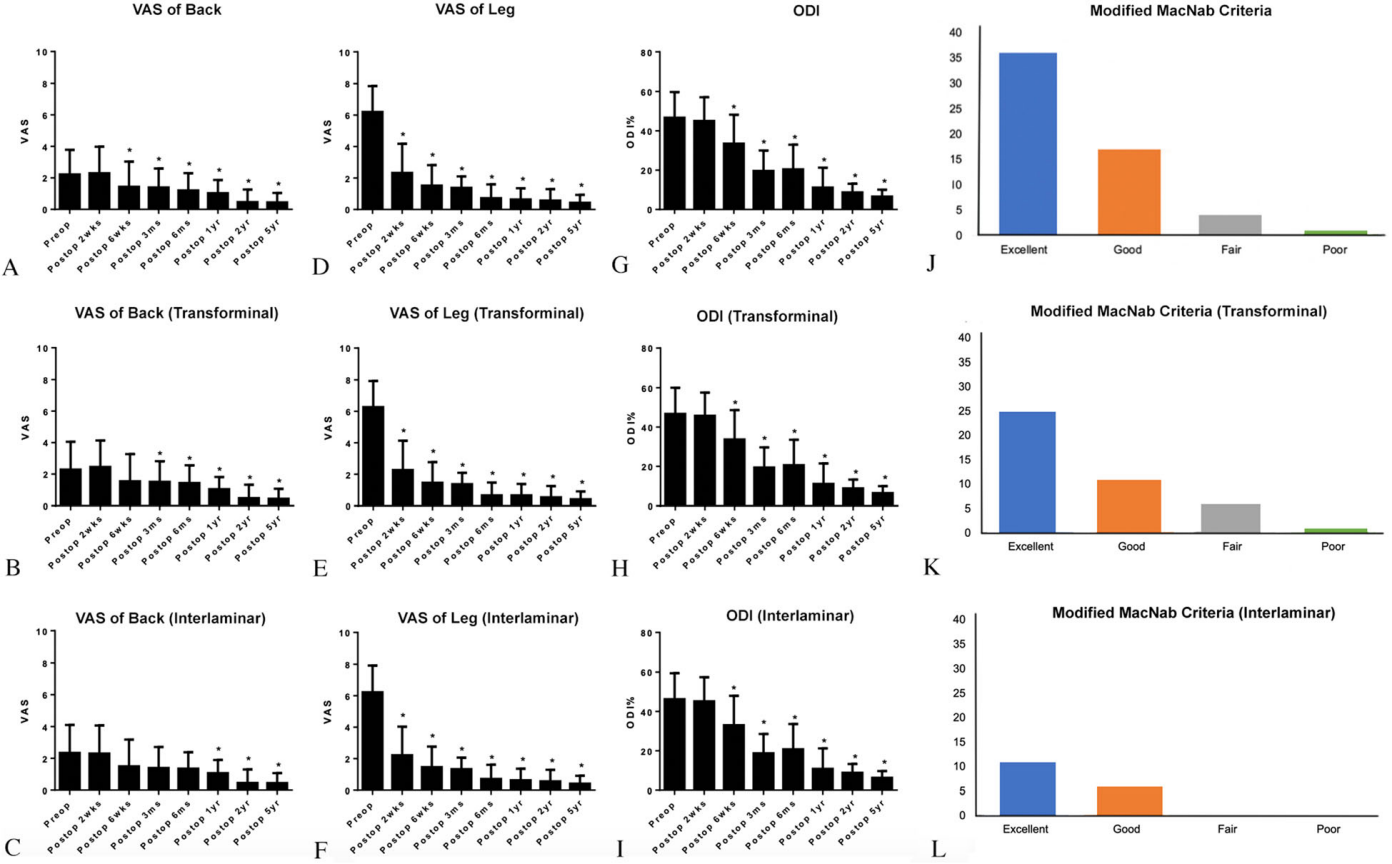
Functional outcomes at each follow-up time. **a** Visual analog scale (VAS) scores for the back. **b** VAS scores for the back in the transforminal approach. **c** VAS scores for the back for IELD. **d** VAS scores of the leg. **e** VAS scores of the leg in the transforminal approach. **f** VAS scores for the back for IELD. **g** Oswestry Disability Index (ODI) scores. **h** ODI scores for the transforminal approach. **i** ODI scores for IELD. **j** Modified MacNab criteria. **k** Transforminal modified MacNab criteria. **l** Interlaminar modified MacNab criteria. VAS: visual analog scale, ODI: Oswestry disability index, * *P* < 0.05

Overall visual analog scale (VAS) scores for back pain showed a significant improvement from 6 weeks after the operation (Table 3, Fig. 8). Patients who underwent TELD exhibited faster improvement (3 months postoperatively) than did those who underwent IELD (1 year postoperatively); however, no significant difference was observed between the two patient groups. The overall and individual VAS scores for leg pain all significantly improved from 2 weeks after the operation. The Oswestry Disability Index (ODI) significantly improved from 6 weeks postoperatively in both the approaches after the operation.

**Table 3:** Satisfaction in patients receiving interlaminar and translaminar endoscopic lumbar discectomy

	Overall (***N***=58)		IELD (***N***=17)		TELD (***N***=41)		
**VAS for back**	Mean	SD	Mean	SD	Mean	SD	***P*** **value**
Preop	2.22	1.57	2.34	1.76	2.29	1.76	0.92
Postop 2 weeks	2.30	1.68	2.30	1.76	2.45	1.69	0.76
Postop 6 weeks	1.44	1.60	1.50	1.68	1.55	1.72	0.92
Postop 3 months	1.40	1.20	1.39	1.32	1.51	1.31	0.75
Postop 6 months	1.20	1.10	1.35	1.03	1.44	1.12	0.78
Postop 1 year	1.03	0.84	1.07	0.82	1.05	0.77	0.93
Postop 2 years	0.46	0.80	0.45	0.85	0.48	0.85	0.90
Postop 5 years	0.45	0.60	0.44	0.63	0.44	0.63	> 0.99
**VAS for leg**	**Mean**	**SD**	**Mean**	**SD**	**Mean**	**SD**	***P*** **value**
Preop	6.21	1.64	2.34	1.70	2.29	1.66	0.92
Postop 2 weeks	2.33	1.85	2.30	1.82	2.45	1.87	0.76
Postop 6 weeks	1.52	1.30	1.50	1.31	1.55	1.31	0.92
Postop 3 months	1.38	0.72	1.39	0.73	1.51	0.73	0.75
Postop 6 months	0.72	0.87	1.35	0.90	1.44	0.82	0.78
Postop 1 year	0.64	0.72	1.07	0.73	1.05	0.73	0.93
Postop 2 years	0.57	0.73	0.45	0.74	0.48	0.71	0.90
Postop 5 years	0.43	0.50	0.44	0.50	0.44	0.50	> 0.99
**ODI**	**Mean**	**SD**	**Mean**	**SD**	**Mean**	**SD**	***P*** **value**
Preop	46.73	13.07	46.24	13.20	46.67	13.36	0.91
Postop 2 weeks	45.00	12.12	45.18	12.25	45.76	11.83	0.87
Postop 6 weeks	33.51	14.71	33.06	14.88	33.73	14.90	0.88
Postop 3 months	19.63	10.38	18.76	9.81	19.46	10.27	0.81
Postop 6 months	20.40	12.57	20.77	12.84	20.66	12.90	0.98
Postop 1 year	11.18	10.14	10.84	10.38	11.11	10.41	0.93
Postop 2 years	8.72	4.41	8.83	4.44	8.86	4.41	0.98
Postop 5 years	6.60	3.53	6.30	3.42	6.46	3.49	0.87
**Modified MacNab**							
Excellent	36		11		25		0.49
Good	17		6		11		
Fair	4		0		4		
Poor	1		0		1		

*IELD* Interlaminar endoscopic lumbar discectomy, *ODI* Oswestry disability index, *TELD* Transforaminal endoscopic lumbar discectomy, *VAS* visual analog scale

The percentage of patients with good to excellent results according to the modified MacNab criteria was 91.3% (53/58 patients). Among the 41 patients who received TELD, the satisfaction rate was 87.8%; five patients exhibited unsatisfactory results. Two patients had a residual disc (1 patient had a repeat TELD the following day, and one patient changed from L4–L5 TELD to L5–S1 IELD during the surgery; Fig. 5). Two patients required an open discectomy due to recurrent disc herniation. One patient (who received IELD) had spinal fusion surgery due to segmental instability after 5 years. The good to excellent result rate was 100% in the 17 patients who underwent IELD.

## Discussion

### Long-term surgical outcomes in high-grade disc migration

After Kambin introduced the posterolateral percutaneous lumbar disc decompression technique in 1973, the use of minimally invasive surgery has become increasingly common [23]. The advancement of specialized tools, such as flexible probes, lasers, and endoscopes, has made FELD highly popular [24]. FELD has been limited to the management of low-grade migrated lumbar disc herniation and has not been used for highly migrated discs, which pose technical challenges to spine surgeons [17]. Lee et al. reported that patients with high-grade migration had a significantly higher incidence of failure (21.1%) than did those with low-grade migration [22]. Recently, various techniques have been developed to treat high-grade migrated discs, and these techniques have shown promising results (Table 2). In our study, we found that TELD and IELD both resulted in satisfactory long-term functional outcomes for high-grade migrated disc herniation.

### TELD for migrated discs

TELD and open discectomy have exhibited similar results in the management of soft high-grade disc migration; moreover, TELD is a safe and effective procedure especially from L1 to L5 [11]. The migratory patterns of the disc fragment are usually restricted by the attachment of the posterior longitudinal ligament, peridural membrane, and midline septum [25]. Thus, to enter the foramen, they remain on the lateral side of the midline [4]. Osman et al. showed that without sacrificing stability, transforaminal decompression can create a considerably larger intervertebral foraminal space compared with posterior decompression (45.5% vs. 34.2% increase) [23]. Furthermore, upward-migrated herniations are common in older patients with associated comorbidities such as diabetes and hypertension, making them ineligible to receive general anesthesia and open surgery [26, 27]. Positioning the patient to achieve hip flexion and low lordosis enlarges the foramen; consequently, the space is sufficiently large for performing TELD without requiring foraminoplasty. However, for zone 1 and zone 4 migration, Kim et al. reported a transforaminal suprapedicular approach with a flexible semirigid curved probe. Curved forceps are extremely useful for the complete removal of very-high-grade disc migration, allowing the surgeon to reach distant sites and remove disc fragments without further bone resection and release soft tissue adhesions [4].

Reamers and endoscopic drills, endoscopic osteotomes, and trepans can help remove the barrier of the pedicle to the disc for highly migrated discs. When performing this step, the surgeon should be extremely careful to prevent neural damage or the significant removal of bony structures leading to lumbar instability. Thus, surgeons are recommended to use endoscopic drills or endoscopic osteotomes to increase precision.

In this study, patients who received TELD had higher VAS and ODI scores than did those who received IELD, probably because TELD caused less damage than IELD through the facet joint during the exploration of the migrated disc. Furthermore, TELD has a shorter operating time than IELD does because TELD requires less bone work and soft tissue management.

The zone with highest number of failed cases of TELD in the present study was in zone 1. The migrated disc at the ipsilateral side was usually blocked by the pedicle unless contralateral TELD was used [13]. The surgeon may be unable to grasp the fragment due to the nonflexible instrument being unable to make an acute turn to reach the area. The contralateral approach enables the surgeon to reach the fragment directly because the angle between the instrument and the migrated fragment is straight. If a bone drill or trephine is available, the use of the translaminar approach [17], transpedicular approach [15, 28], or keyhole procedure for directly targeting the migrated disc can result in a satisfactory outcome. Foraminoplasty is needed to access high-grade migrated disc herniations for multiple reasons. First, lumbar herniation occurs most frequently at lower levels. The diameter of the intervertebral foramen decreases in the lumbar area, from cranial to caudal. Narrowing may result from degenerative changes due to the hypertrophy and the overriding of facets and the thickening of the LF. For adequate decompression, the anterior epidural space must be reached under direct visual control. Enlarging the foramen by undercutting the superior articular facet can facilitate reaching the epidural space and ensure the adequate exposure or complete removal of the fragment [29].

### IELD

In this study, IELD was more effective than TELD for L4–L5 high-grade downward and L5–S1 high-grade upward and downward migration discs, except in failed cases. Axillary herniated discs can be easily removed using IELD. The S1 nerve root exit at the L5–S1 level disc space is unique. The angle between the S1 root and thecal sac allows access to the axillary portion of the S1 nerve root. An increase in the angle between the root and thecal sac in axillary disc herniation increases the working space for the cannula without damaging the root. IELD can directly access the axillary herniated disc and remove the disc fragment with minimal manipulation of the neural structure. However, with the use of TELD, incomplete decompression or a remnant disc is possible. The posterior longitudinal ligament must be cut to retrieve the dorsally migrated disc fragment [19].

As shown in Table 4, after gaining the in-depth knowledge of surgical anatomy and ensuring strict adherence to technical guidelines, endoscopic surgery does not yield poor outcomes when performed for managing high-grade disc migration. Using an accurate approach for the proper indication remains the most crucial point. Doctors must gain in-depth knowledge of surgical anatomy and ensure strict adherence to technical guidelines; familiarity with IELD and TELD is particularly important. TELD is preferred for shoulder-type disc herniation, centrally located disc herniation, and recurrent disc herniation. IELD is preferred for axillary-type disc herniation and migrated discs, particularly high-grade disc migration and disc herniation.

**Table 4:** Literature review of full endoscopic spine surgery to manage high-grade migrated lumbar disc herniation

Name	Surgical Technique	Details	Patients	Modified MacNab	VAS Preop	VAS Postop	ODI Preop	ODI Postop	Recurrent Herniation
Gun Choi 2008 [4]	PELD with foraminoplasty under local anesthesia	Anesthetizing methods/positioning: Local anesthesia with the patient in prone position Site of annular puncture • L4–L5 and below: Medial pedicular line • L3–L4 and above: Midpedicular line Inclination of the Needle Trajectory • Approximately 30° with the lower or upper endplate Down-migrated herniation • Remove undersurface of the articular process • Use endoscopic drill with a round diamond burr tip to removal bony part • Allows for placement for endoscope in anterior epidural space • Ligamentum flavum, fibrotic bands, part of anulus removed using Holmium: Yttrium–Aluminum–Garnet (YAG) laser • Intermittently blocking the irrigation fluid outflow allows traversing nerve root to move freely: Confirms complete decompression Up-migrated herniation • Use of round-ended cannula • Placement of cannula at the lower part of the disc • Upward shifting with twisting motion till the exiting root is partially visible • Release of the foraminal ligament and the Ligamentum flavum using laser • Removal of the exposed ruptured fragment with forceps	59	91.4% of patients experienced satisfactory outcome Good: 37 patients (63.8%) Fair: 16 (27.6%) Poor: 4 patients (6.9%) Poor: 1 (1.7%)	8.01	1.56	61.6	10.76	2
Hyeun Sung Kim 2009 [14]	Endoscopic transforaminal suprapedicular	Anesthetizing methods: Favor local anesthetic Skin entry approximately 8–12 cm from the midline Removal of the superior margin of the pedicle Spondylosis in the upper margin of the lower vertebrae • Traversing nerve root is exposed • Remove ruptured disc material Inferior migrated ruptured material below the traversing nerve root • Be aware of not to injure the traversing nerve root • Semirigid flexible curved forceps to pull the disc material • Bleeding may occur. Use bipolar coagulation and saline irrigation • Check for blue-stained disc material • No stain implies successful removal	53	N/a	Leg: 9.32 ± 0.43	Leg: 1.78 ± 0.71	79.82 ± 4.53	15.27 ± 3.82	N/a
G. Choi 2010 [19]	Percutaneous Endoscopic Lumbar Herniectomy for high-grade down-migrated L4–L5 disc through an L5–S1 IELD	Anesthetizing methods/positioning • Lateral decubitus position with the affected side upwards • Conscious sedation Skin entry point • Used 1% lidocaine • 0.5-mm to 0.7-mm skin incision Herniectomy was performed using various grasping forceps and side-firing holmium-YAG laser Flexible tip of the Ellman radiofrequency probe can be used for hemostasis and tissue dissection.	4	N/a	Back: 3.75 Leg: 8.5	Back: 1.75 Leg: 0.75	65%	3%	N/a
Kyeong-seong Yeom 2011 [13]	Full endoscopic contralateral transforaminal discectomy	Anesthetizing methods/positioning • Prone position on a radiolucent operating table • Epidural anesthesia Skin entry site: L3–L4 and L4–L5 levels: Dorsal portion of the facet joint of index level on the lateral view of the C-arm. Inject a mixture of indigo–carmine and radio-opaque dye Endoscope was inserted to the anterolateral side of facet joint Foraminoplasty was done provided enough working space Using C-arm guidance to confirm facet articulation Insert trephine removed anterolateral bony portions of the facet joint • Unsuccessful: Micro-osteotome under endoscopic visualization was used for foraminoplasty Explore site between the posterior longitudinal ligament and the dural sac. - Protect the dural sac by turning the working sheath ventrally facing the posterior longitudinal ligament	12	Excellent: 10 Good: 2	Back: 6.8 Radicular pain: 8.2	Back: 1.5 Radicular pain: 1.4	N/a	N/a	N/a
Jianwei Du 2016 [17]	Translaminar approach	Anesthetizing methods/positioning • Prone position with mild flexion of the hips and knees • Local anesthesia Target site of puncture: 8 to 10 mm from midline Building of the working cannula • Guide wire inserted through needle • Insert endoscope with an eccentric 2.7-mm working Drilling bony tunnel in the lamina • Expose bony surface of lamina with endoscopic forceps and a flexible radio frequency probe • Trepan was used to mark the site for drilling • Round diamond burr tip was used to remove the bony portion of the site • Diameter of the tunnel approximately 8 mm Removal of the migrated herniation • Use 90-degree angle blunt hook to explore the canal and the ventral aspect of the dural sac • Use endoscopic forceps to remove the migrated herniation through the lateral aspect of the dura	7	N/a	7.6 ± 0.8	1.3 ± 0.8	61.6	8.4	0
Yong Ahn 2004 [3]	Standardized technique of transforaminal PELD	Anesthetizing methods/positioning • Local anesthesia with conscious sedation • Midazolam (0.05 mg/kg) intramuscularly and fentanyl (0.8 g/kg) • Prone positioned on a radiolucent table. Direction-oriented transforaminal TELD • Entry point: 10–15 cm lateral to the midline • Discography with indigo–carmine Intradiscal and annular release • Release of annular anchorage • Intradiscal subannular debulking • Use grasping forceps, radiofrequency bipolar ablator, and side-firing laser for intradiscal release • Use annulus scissors to cut outer layer of the annulus and the annulus posterior longitudinal ligament Epiduroscopic fragmentectomy using navigable instruments • Semiflexible forceps, articulating forceps, and flexible curved probe is for complete removal • Ventral decompression can increase working space • Removed in one piece or in multiple pieces	13	Excellent: 4 patients (30.8%) Good: 7 patients (53.8%) Fair: 1 patient (7.7%) Poor: 1 patient (7.7%)	7.86 ± 1.28	6 weeks: 2.54 ± 1.51 1 year: 1.85 ± 1.07	84.92 ± 6.36	6 weeks: 27.83 ± 7.34 1 year: 17.54 ± 13.40	N/a
Jinwei Ying 2016 [29]	Transforaminal PELD Contralateral transforaminal PELD Interlaminar PELD	Anesthetizing methods/positioning • Prone position • Local anesthesia and sedation Transforaminal PELD • Entry point 10–13 cm from the midline • Mixture of methylene blue and Iohexol (2 mL) • Partial pediculectomy was done if fragment of disc was blocked by the pedicle or more space was needed for manipulation Interlaminar PELD • 18-gauge spinal needle was inserted into the disc with the conventional IELD • 2 mL mixture of methylene blue and iohexol for discography • Partial medial laminectomy can be performed if view is blocked Contralateral Transforaminal PELD • Entry point approximately 14 cm from the midline • An 18-gauge spinal needle was introduced into the disc under fluoroscopic guidance • A mixture of methylene blue and Iohexol (2 mL) for discography • Dyed migrated disc fragment was observed • MRI was performed 24 h after surgery confirm complete decompression	73	Transforaminal PELD Excellent: 14 Good: 13 Fair: 4 Poor: 0 Contralateral transforaminal PELD Excellent: 8 Good: 6 Fair: 0 Poor: 1 Interlaminar PELD Excellent: 15 Good: 10 Fair: 2 Poor: 0	Transforaminal PELD Back: 5.8 Leg: 7.2 Contralateral transforaminal PELD Back: 5.5 Leg: 6.5 Interlaminar PELD Back: 5.4 Leg: 7.2	Transforaminal PELD Back: 2 Leg: 2 Contralateral transforaminal PELD Back: 1.9 Leg: 2.1 Interlaminar PELD Back: 2.1 Leg: 2.3	Transforaminal PELD 55 Contralateral transforaminal PELD 57 Interlaminar PELD 55	Transforaminal PELD 18 Contralateral transforaminal PELD 14 Interlaminar PELD 13	N/a
Chi Heon Kim 2016 [20]	TELD Percutaneous endoscopic interlaminar discectomy	Anesthetizing methods/positioning • Prone position • General anesthesia Superior migration • Interlaminar window at the same level of the disc herniation Inferior migration • Interlaminar window at a lower level than the disc Entry point • Sagittal CT scan at midway between the medial margin of the lamina and the spinous process • Extension line was drawn to the skin • Point of intersection between the extension line and skin was the entry point Enlargement of laminar window • Superior migration: not needed • Inferior migration: needed Discography • Posterolateral approach using indigo carmine mixed with radio-opaque dye Ligamentum flavum was opened or split • Compromised more than 50% of the anterior–posterior diameter of the spinal canal: Open Ligamentum flavum • Less than 50%: Ligamentum flavum was split Identify thecal sac and root • Remove disc material	18	Excellent: 12 Good: 3 Fair: 2 Poor: 1	Trunk: 6.1 ± 2.5 Limb: 7.5 ± 1.7	Trunk: 2.8 ± 1.8 Limb: 2.1 ± 2.0	25.7 ± 9.0	8.4 ± 6.1	0
Guntram Krzok 2016 [15]	Transpedicular Lumbar Endoscopic Surgery	Anesthetizing methods/positioning • Lateral decubitus position • Local anesthesia and intravenous sedation Entry point • L3: 10 cm lateral from the pedicle • L4: 11 cm lateral from the pedicle • L5: 12 cm lateral from the pedicle Insert a 25-cm; an 18-gauge needle was at the lateral pedicle between vertebral body and transverse process • Confirm with fluoroscopy Replace needle with K wire • Insert dilators of 4 and 8 mm to the pedicle • Removed dilator and insert a Yamshidi needle into the pedicle with fluoroscopic guidance Yamshidi needle insertion • Middle of the pedicle in the AP and lateral views • Loss of resistance and occasionally leg pain of the patient means the pedicle has been penetrated • Replaced Yamshidi needle with 2-mm K wire and disposable bone drill of 4 mm Small bone hole is then increased in size increasing diameters of drills or reamers to 8 mm 7.2-mm tubular retractor is inserted Mixture of contrast medium (Solutrast 3 mL) and Toluidine blue dye (0.1 mL) • Remove the blue-stained disc sequestration with bendable forceps	21	N/a	8.1	1.3	N/a	N/a	N/a
Xinbo Wu 2016 [8]	TELD Two-channel technique	Anesthetizing methods/positioning • Prone • Local anesthesia with lidocaine (1%) Surgical puncture point • 10 cm from the midline for L3–L4 segment • 11–14 cm from midline for L4–L5 segment Lateral fluoroscopy confirmed the needle positioned above the vertebral foramen	22	Excellent: 14 Good: 6 Fair: 2	Back: 7.82 ± 0.96 Leg: 8.59 ± 1.05	Back: 1.14 ± 0.71 Leg: 0.95 ± 0.72	71.18 ± 7.90	16.91 ± 4.13	1
Kyung-Chul Choi, 2016 [5, 9]	Epiduroscopic Laser Neural Decompression (ELND) for Down-migrated Disc Herniation	Anesthetizing methods/positioning: • Local anesthesia • Prone position Underwent PELD via the transforaminal route for removal of a paracentral extruded disc Opening of the epidural space between the extruded disc and traversing nerve root Cannula location • 25° trajectory angle • Between the spinous process and medial pedicle line on anteroposterior radiography Herniated disc was removed using endoscopic forceps Using bipolar and endoscopic scissor, release the outer annulus and posterior longitudinal ligament ELND was done via sacral hiatus for removal of the down-migrated disc by using a Holmium: YAG laser The flexible epidural fiber optic catheter system was inserted through the sacral hiatus With fluoroscopic guidance, catheter went up to the pedicle along the ventral surface of the epidural space Differentiate nerve root under epiduroscopic view and vaporized with laser (5 J at 5–10 Hz) Performing PELD remove free fragments with forceps	1	N/a	N/a	N/a	N/a	N/a	N/a
Hyeun Sung Kim 2018 [14]	Percutaneous endoscopic transforaminal lumbar discectomy Percutaneous endoscopic interlaminar lumbar discectomy	Anesthetizing methods/positioning • Prone position Spine needle insertion point • Toward the lowest part and most dorsal part of disc space • Infltrated with 7–10 mL 1% lidocaine followed by epinephrine mixed 2–3 cc 1.6% lidocaine, 3–5 min after the first injection Discography using a contrast mixture consisting of 6 mL iohexol dye and 1 mL indigo–carmine Tapered cannulated obturator inserted over the guide wire and advanced into the foraminal space Internal disc decompression Remove tissue around the pedicle Perform suprapedicular circumferential opening technique • Drilling ventral part of superior articular process, the upper part of pedicle that builds the suprapedicular notch, upper-posterior margin of the lower vertebra Use semirigid flexible curved probe and forceps to hook and pull the disc material out	98	Poor: 1 (1.0%) Fair: 3 (2.9%) Good: 54 (51.9%) Excellent: 46 (44.2%)	Leg: 7.13	Leg: 1.95	54.67 ± 7.52	24.50+ 6.45	N/a
Quillo-Olvera 2018 [28]	PELD transpedicular approach	Anesthetizing methods/positioning • Prone with hips and knees in flexion • Local anesthesia with conscious sedation Skin entry • 10 cm lateral from the midline for the L3 pedicle • 11 cm lateral from the midline for the L4 pedicle • 12 cm lateral from the midline for the L5 pedicle Skin is infiltrated with 1% lidocaine An 18-gauge spinal needle is advanced and placed on the lateral wall of the pedicle, behind the transverse process The spinal needle is replaced with K wire Insert obturator and the tip should be placed on the lateral wall of the pedicle • Right pedicle at 3 o’clock, and for the left pedicle at 9 o’clock 25° rod-lens endoscope of 6.3-mm outer diameter is advanced to visualize the lateral wall of the pedicle Create a transpedicular tunnel Remove a thin layer of cortical bone from its medial wall with endoscopic Kerrison punch • Endoscope is advanced through the tunnel to visualize the migrated disc herniation directly Endoscopic nerve hook used to confirm that the herniated disc has been completely removed	N/a	N/a	N/a	N/a	N/a	N/a	N/a

*IELD* Interlaminar endoscopic lumbar discectomy, *ODI* Oswestry disability index, *PELD* Percutaneous endoscopic lumbar discectomy, *TELD* Transforaminal endoscopic lumbar discectomy, *VAS* visual analog scale

A limitation of this study is the retrospective nature of data collection. However, the prospective spine registry effectively collected postoperative function scores, which reduced missing data. In addition, selection bias due to loss to follow-up remains a concern in this study. However, we had a follow-up rate of > 80% for patients with high-grade migrated discs, which reduced the bias. Furthermore, this study is limited by its small sample size, and the power of the study in comparing TELD and IELD was not evaluated. Additional studies comparing TELD and IELD for high-grade disc migration are needed. Another limitation of this study is long patient hospitalization due to affordable health care expenses, which may not be comparable to other studies.

## Conclusion

FELD has a high success rate for the management of high-grade disc migration and disc herniation. TELD is effective and safe for patients with high-grade disc migration from L1 to L5. In patients with L4–L5 and L5–S1 high-grade upward and downward disc migration, IELD is a favorable option providing high patient satisfaction.

## Data Availability

The data supporting our findings can be found in the article.

## Abbreviations

FELD: Full-endoscopic lumbar discectomy
TELD: Transforaminal endoscopic lumbar discectomy
IELD: Interlaminar endoscopic lumbar discectomy
CT: Computed tomography
MRI: Magnetic resonance imaging
LF: Ligamentum flavum
VAS: Visual analog scale
ODI: Oswestry Disability Index
